# Hair cortisol and self-perceived stress in adolescents with functional somatic disorders – a comparison with general population data

**DOI:** 10.1192/j.eurpsy.2022.1086

**Published:** 2022-09-01

**Authors:** R. Nyengaard, K. Kallesøe, M. Rimvall, E. Ørnbøl, E.M. Olsen, V. Wyller, C. Rask

**Affiliations:** 1 Psychiatry, Aarhus University Hospital, Research Unit, Department Of Child And Adolescent Psychiatry, Aarhus N, Denmark; 2 Aarhus University, Department Of Clinical Medicine, Aarhus C, Denmark; 3 Mental Health Services, Capital Region of Denmark, Child And Adolescent Mental Health Centre, Hellerup, Denmark; 4 Psychiatry Region Zealand, Department Of Child And Adolescent Psychiatry, Roskilde, Denmark; 5 Aarhus University Hospital, Research Clinic For Functional Disorders And Psychosomatics, Aarhus N, Denmark; 6 Bispebjerg & Frederiksberg Hospital, Centre For Clinical Research And Prevention, Copenhagen N V, Denmark; 7 Psychiatric Center Ballerup, Outpatient Clinic For Eating Disorders In Adults, Ballerup, Denmark; 8 Akershus University Hospital, Department Of Pediatric And Adolescent Medicine, Nordbyhagen, Norway; 9 University of Oslo, Institute Of Clinical Medicine, Oslo, Norway

**Keywords:** hair cortisol, functional somatic disorder, Stress, adolescent

## Abstract

**Introduction:**

Functional somatic disorders (FSDs) are characterized by persistent and disabling physical symptoms that cannot be attributed to well-defined somatic disorders. In adolescents, the prevalence is around 4-10%. Evidence from adult populations suggests that cortisol plays a role in the development and perpetuation of FSDs, but little is known regarding adolescents. As cortisol accumulates in hair over time, hair cortisol concentration (HCC) is a promising new biomarker for long-term physiological stress. Moreover, adult studies have found associations between HCC levels and self-perceived stress.

**Objectives:**

To compare HCC levels between adolescents with severe FSDs and adolescents from the general population. Furthermore, to investigate the association between HCC and self-perceived stress.

**Methods:**

The data are retrieved from two projects: the AHEAD trial, including 91 15-19-year-old adolescents diagnosed with a severe FSD, and the Copenhagen Child Cohort 2000 (CCC2000), including data on 1455 16-17-year-old adolescents. Hair samples were collected for HCC analysis, and web-based questionnaires were used to asses self-perceived stress. Functional somatic symptoms were assessed with the Bodily Distress Syndrome (BDS) checklist.

**Results:**

The data have been collected and will be analysed and presented at the congress.

**Conclusions:**

This study can contribute with knowledge about the potential role of cortisol in FSDs in adolescents, and whether self-perceived stress can be used as a marker for physiological stress measured by HCC. Treatments for adolescents with FSDs still need to be improved. The current study may help to understand whether future treatment strategies should include a greater focus on stress management.

**Disclosure:**

No significant relationships.

